# Assessment of street-level greenness and its association with housing prices in a metropolitan area

**DOI:** 10.1038/s41598-023-49845-0

**Published:** 2023-12-19

**Authors:** Sihyun An, Hanwool Jang, Hwahwan Kim, Yena Song, Kwangwon Ahn

**Affiliations:** 1 Department of Industrial Engineering, Yonsei University, Seoul, 03722 South Korea; 2 Center for Finance and Technology, Yonsei University, Seoul, 03722 South Korea; 3 Department of Finance, Accounting and Risk, Glasgow Caledonian University, Glasgow, G4 0BA UK; 4 Department of Geography, Chonnam National University, Gwangju, 61186 South Korea

**Keywords:** Environmental economics, Environmental impact, Environmental impact

## Abstract

Rapid global urbanization has made environmental amenities scarce despite their considerable advantages, ranging from aesthetics to health benefits. Street greenness is a key urban environmental amenity. This study developed a green index as an objective measure of greenness using street view images and assessed its predictive power along with that of other environmental amenities for metropolitan housing prices. Spatial interpolation was used to transform point data into areal data, enabling effective analysis of a dataset covering an entire metropolis. A series of hedonic models revealed that (1) street greenness is significantly and negatively associated with housing prices, (2) a traditional greenness indicator and the green index provide complementary information, indicating that they could be used for different purposes, and (3) environmental amenities, in general, demonstrated significant relationships with housing prices. Our analysis strategy including spatial interpolation can be widely employed for studies using different types of data. The findings demonstrating a complementary relationship between our two greenness indicators provide valuable insights for policymakers and urban planners to improve street-level greenness and green accessibility. Considering the significance of environmental amenities, this study provides practical approaches for executing sustainable and healthy city development.

## Introduction

The world has been rapidly urbanizing since the Industrial Revolution, which has expanded the built environment at the expense of the natural environment in urban areas. As the natural environment and environmental amenities have become scarcer in urban areas, they have earned more appreciation and the importance of the correlation between greenness, sustainability, and property value has been highlighted across investors and residential communities. For instance, real estate investors in the US seek to target investments toward more sustainable properties with green spaces, as they begin to consider sustainable initiatives with support of the Biden administration^1^. Notably, a Los Angeles law mandates a certain amount of open green space when constructing six or more residential units^2^.

It has been well documented that surrounding environmental amenities provide various benefits to neighborhoods and command financial premiums. Furthermore, residents in green areas are found to be more physically and mentally healthy^3–5^. Green streets and nearby parks encourage people to walk more and stay active and mitigate pollution, carbon footprints, and urban heat island effects, providing a healthier environment in which to work and live^6–8^. In that context, Kruize et al.^9^ noted ‘triple win’ of green space, which highlighted the potential of green spaces to create environmental sustainability, health, and health equity. The appreciation of natural environmental amenities such as green spaces, planted streets, and green walls could be partly capitalized in property prices^10–12^. Similarly, such preferences may encourage people to willingly move to areas with more environmental amenities despite the higher cost^13^.

Empirical studies examining the benefits of urban environmental amenities have often struggled to identify appropriate variables that represent specific landscape components and spatial scale. However, recent advances in information technology and the broad availability of urban data provide realistic and robust means of assessing urban landscapes. In particular, Google Street View (GSV) images that are available online are considered as a novel data source that researchers have employed in various empirical studies^14–16^.

The majority of previous studies has not sufficiently explored the relationship between visual greenness of street-level and proximity to green space. In particular, the linkage between the visual quality of urban greenness and proximity to green space is often overlooked in urban planning, potentially resulting in unbalanced development of the ecological environment. In this context, we provide a novel and informative perspective regarding whether visual greenness and proximity to green space have a substitutional or complementary relationship. This study includes other environmental amenities along with built environmental components and, so that our results can provide a more comprehensive understanding of urbanites’ appraisal for better natural environment after controlling other factors. Therefore, our findings can be used as a guideline for advancing ecological transitions to achieve urban and environmental sustainability.

This study explores the potential for combining readily available street images and other environmental amenity variables to predict housing prices, specifically investigating three questions. (1) Is greenness at the street-level associated with housing prices? (2) If so, is street-level greenness an alternative or complementary to traditional green amenity variables in predicting housing prices? (3) How are environmental amenities related to housing prices in a metropolitan area?

We take a three-step approach to addressing these questions. First, we measure the greenness of a whole city using street images. We then calculate neighborhood greenness using the point-level images and finally, explore its association with housing prices, along with other explanatory variables.

## Literature review

### Local environmental amenities and property prices

Living close to desirable environmental amenities or natural resources is considered to provide residents with numerous welfare benefits, including opportunities for recreation, leisure, social interaction and integration, air purification and cooling, noise reduction, biodiversity preservation, and improved mental and physical health^7,17,18^. Numerous studies have examined the value of environmental amenities. In real estate research, such efforts have involved in measuring the impact of environmental amenities on property prices^12^.

The literature has indicated that environmental amenities could be a key factor determining housing prices. For instance, Gibbons et al.^17^ analyzed one million housing transaction records in England and reported that properties near green space and/or water command a considerable premium. A pleasant view, particularly one overlooking water or green space, was also found to add financial value to real estate properties^10,11,19^. After reviewing 30 studies, Crompton^20^ concluded that proximity to parks generally increased property values, although the impact would depend on the type of park and its distance from a residence. Furthermore, in the tourism sector, a phenomenon called “*Albergo Diffuso (AD, scattered hotels)*” was born a few decades ago primarily in Italy^21^. It represents an example of how to build a business into the historical villages avoiding massive alteration of existing landscapes but enhancing what the communities have. This concept enables to add value to the business and communities whilst keeping the original landscape and environment^22^. Generally, it appears that desirable natural amenities have positive effects on property prices and communities as well^23–26^.

Conversely, evidence has revealed that not all environmental amenities contribute positively to property values. Panduro and Veie^27^ classified green assets into eight categories and found that the presence of green space buffering unattractive land was negatively associated with housing prices in an urban area. Similarly, Sharma^28^ revealed different associations between different green amenities and residential land prices in mountain counties in Colorado, wherein green amenities were sometimes found to have no significant or even negative impacts on property prices^18,29^. Such negativities can be characterized as sources of allergic reactions to pollen or habitats for disease vectors, especially in residential areas^30^. Moreover, environmental amenities that are appreciated in one area could be considered negative in other areas; thus, financial impacts can depend on the local context^31^. Such findings seem to contradict the general consensus on the association between environmental amenities and property prices, emphasizing the need for additional empirical work, more sophisticated analyses, and careful interpretation.

### Measuring greenness

Greenness, particularly urban greenness, has long attracted attention in both academic circles and public practice. Over the past few decades, the increasing availability of various datasets, including massive image data, has enabled analyses using more sophisticated and scientifically rigorous measurements. Traditionally, quantitative proxies of urban greenness were used, such as the distance to green spaces or the area of green space in land-use databases^32–35^. Qualitative methods have also been employed such as surveys, interviews, and professional audits^12,36^.

The normalized difference vegetation index (NDVI) is a commonly used measure of urban greenness^37–39^ that is calculated using satellite images featuring various types of greenery. The NDVI includes pixel-wise data that are not affected by artificial boundaries such as administrative units, enabling wide applicability. However, because it uses satellite imagery, the NDVI captures greenness at the canopy level and cannot capture the vertical dimension^40^. Measured in this manner, the level of greenness does not necessarily reflect the experience of the general public and often overestimates the extent of green areas that are accessible to residents in daily life^8,41,42^. In short, the NDVI does not consider three-dimensional greenness as NDVI calculates the amount of greenness from an overhead view and only presents a two-dimensional indicator^8^. This measurement differs from the actual amount of greenness that is observed by pedestrians^43^; therefore, it is not an appropriate indicator for reflecting pedestrians’ and residents’ exposure to greenness in everyday life.

Recent developments in smart technology provide various sources of big data related to local landscapes, including GSV. GSV images are now available for a considerable number of cities and have become a popular big data source for academic research^8,15^. For instance, Li et al.^41^ measured street greenness using GSV images and documented that higher income groups tended to prefer to live in green neighborhoods. Additional studies found that a street greenery index calculated from GSV images had a positive association with commercial buildings’ prices and rents^16^ and walking behavior in an urban area^8^. Rzotkiewicz et al.^15^ reviewed 54 studies using GSV and concluded that it is a time- and cost-efficient data source. Not all areas are well covered, missing imagery is more common in less developed and rural areas; thus, GSV can serve as a potentially reliable and promising data source for a case study focusing on urban areas in a developed country.

### Green visibility and accessibility in relation to property

We address green visibility as a proxy for the degree of daily exposure to urban greenness and green accessibility as the proximity to green spaces, and subsequently explore recently published studies addressing the significance of greenness in relation with property values. Table 1 summarizes examples identified in decent academic journals. The existing literature can be categorized in terms of the variables used in the studies’ investigations (e.g. green visibility, green accessibility, and both green indicators).

**Table 1:** Examples of recently published academic literature on urban greenness and property prices.

Authors (year)	Survey area (period)	Property type	Data coverage	Green type	Sign
Green visibility					
Ki and Lee (2021)^8^	Seoul (2014–2018 period)	Residential (apartments)	Google street view	Street greenness	Positive
Yang et al. (2021)^16^	Manhattan, New York (2010–2017)	Commercial (office buildings)	Google street view	Street greenness	Positive
Dou et al. (2023)^44^	Shanghai (November 2018)	Residential (apartments)	Baidu street view	Street greenness	Positive
Qiu et al. (2023)^45^	Shanghai (2019)	Residential (apartments)	Baidu street view	Street greenness	Positive
Sachs et al. (2023)^46^	Baltimore, Maryland (2009–2011)	Residential	Tree canopy from Oak Ridge National Laboratory	Urban greenness	Positive
Teo et al. (2023)^47^	Singapore (1990–2019)	Residential (apartments)	Land cover fraction map using google earth engine	Urban greenness	Positive
Green accessibility					
Chen et al. (2020)^48^	Shanghai (April 2017)	Residential	Amap (China’s popular navigation map)	Time consumption from a community to the nearest green space	Positive
Doan (2023)^49^	Hanoi (2018–2019)	Residential	Questionnaire survey	Distance to green space	Insignificant
Green visibility and accessibility					
Wu et al. (2022)^26^	Shenzhen	Residential	Baidu steet view	Street greenness	Positive
			Anjuke	Distance to the nearest community, city, and natural parks	Positive
Dai et al. (2023)^50^	Haifa (1998–2016)	Residential	ArcGIS visibility toolset	Natural greenness	Positive
			Survey of Israeli national buildings layer	Distance to natural green area	Negative
Zhang (2023)^51^	Nanjing	Residential	Baidu street view	Street greenness	Positive
			Baidu satellite map	Distance to the nearest entrance of the structured green space	Positive

Green visibility studies have primarily used GSV images and land cover data. Those that have calculated greenness using GSV images have assumed measures standing for residents’ level of exposure to green^8,16,26,44,45,51^. If the images were distributed evenly and densely, the interpretation of measures was deemed to be correct; however, GSV images often suffer from uneven spatial distribution (even in metropolitan areas), leaving certain areas without any images or with only few images. This can be worse when streets are sparse or on the outskirts of a city; thus, properties located near mountains, hills, and forests are more likely to have a small number of images or no images related with neighborhood greenness. Most of these studies did not address this limitation in detail and it remains a relevant issue that must be properly handled. In contrast, studies using land cover data consider natural trees, shrubs, grass, and forests as natural greenness and generally do not suffer from lack of data over various spaces; however, as noted previously, the greenness indicators used are far from street-level and do not reflect pedestrian’s perspective.

Another line of studies has employed the distance to green space as a proxy for green accessibility. Dai et al.^50^ documented that accessibility to natural greenness is negatively associated with housing prices due to the incursion and threat of wildlife. Doan^49^ reported that the relationship between distance to green space and housing price is insignificant. Meanwhile, a majority of studies have demonstrated that green accessibility has a positive impact on housing prices^26,48,50,51^. Although proximity to green space could be a significant factor of residential property values, no research has examined the impact of green accessibility on property prices after controlling for the green visibility.

Prior studies have provided considerable insights regarding data sources, methodologies, and interpretation for understanding the relationship between greenness and property values; however, our study contributes to the literature by filling the gaps noted above. Access to urban greenness can be an indicator of whether the spatial distribution of green spaces matches nearby residents’ demand^52^. Meanwhile, different degrees of green visibility may have an impact on residents’ perception to urban greenness^53^, including physical, mental, and health benefits^54,55^. Accordingly, we argue that two greenness indicators would function differently; thus, the lack of exploration regarding the relationship between two greenness indicators must be further addressed. In this process, potential errors that occurred due to imperfect image distribution must be addressed. To that end, we employ a spatial interpolation strategy in this study to scientifically and rigorously develop greenness indicators that realistically demonstrate the relationship between the two greenness indicators in a concrete way.

## Data and methodology

### Study scope and data

**Figure 1:**
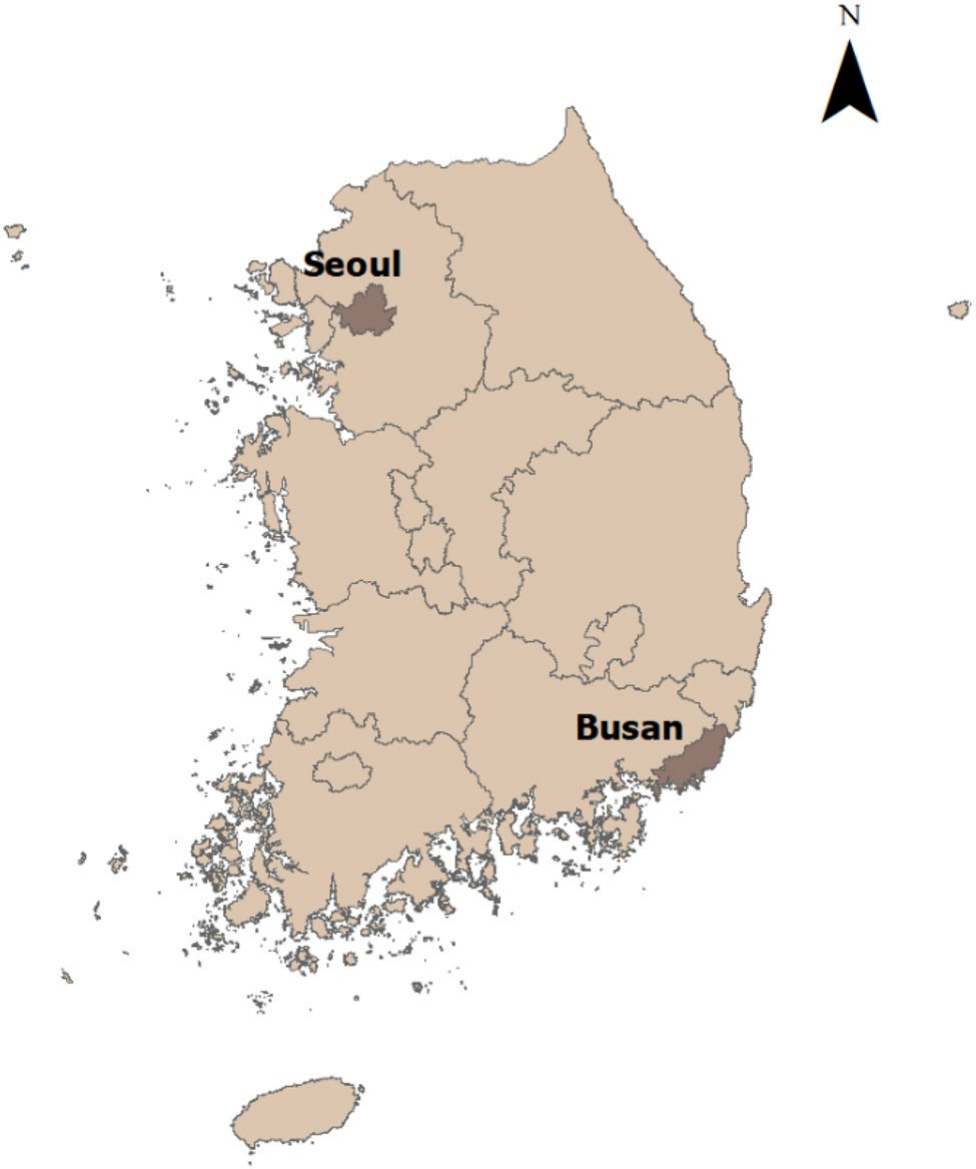
Case study area: Busan. This map was created by using ArcMap software (https://desktop.arcgis.com/en/documentation/), version 10.3.0.

The case study site is Busan, located at the southeastern end of the Korean peninsula (Fig. 1). It was chosen because it is a metropolitan city with a large population of approximately 3.4 million in 2022^56^ and has a large volume of housing sales transaction records. Ahn et al.^35^ noted that Busan had the largest volume of transaction records among metropolitan areas in South Korea, excluding the Greater Seoul area. Second, unlike other metropolitan areas in South Korea, the city has a variety of natural amenities, including a long seafront, hills, and mountains. Finally, Busan attracts a large number of tourists. The city had long been recognized as a heavy industrial center but recently became a tourist city. Its location as a nearby waterfront, history of serving as a haven during wartime, and various natural environments in the city are key factors for this change. As such, the environmental diversity in this city makes it possible to control for other environmental variables when assessing the impact of street greenery on housing prices.

**Figure 2:**
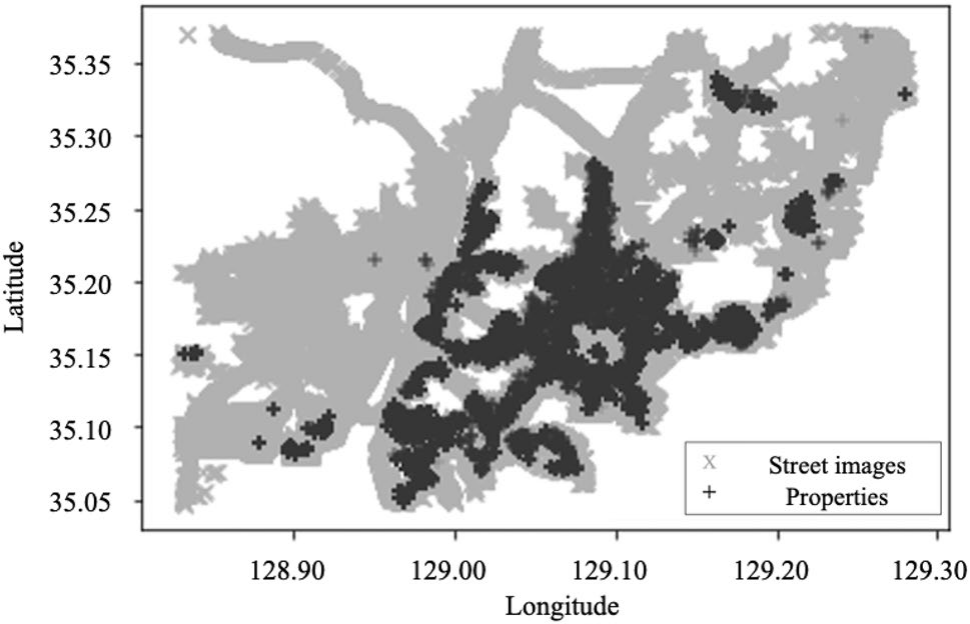
Spatial distribution of property transactions and GSV images in Busan. This map was created by using Matplotlib library (https://matplotlib.org/stable/index.html), version 3.7.1.

The green index was derived from GSV 360° panorama images, which are obtained with the GSV download tool (https://svd360.istreetview.com). GSV provides continuous panoramic street views, providing a complete virtual representation of the landscape from the pedestrian viewing angle. GSV images were acquired covering the whole case site, totaling 409,390 images (Fig. 2) taken between 2017 and 2018. We removed outliers where natural greenery is difficult to discriminate, such as in playgrounds with green paintings and tunnels. These removals resulted in a dataset of 306,425 images that were deemed suitable for precise analysis of the impact of natural greenery.

One of the key variables in this study is property value. In Korea, the Ministry of Land, Infrastructure and Transport (MLIT) provides transaction data for all housing properties online. MLIT transaction data cover various types of housing properties; however, we only use apartment transaction data, as these are the only data type that includes full addresses^35^. The MLIT apartment transaction records include transaction prices, property addresses, and a few property characteristics such as parking facilities, year of construction, and heating type. We obtained and cleaned all apartment transactions from 2018 and 2019, yielding 52,644 transactions for analysis after removing observations without location information.

Data for other variables were obtained from public data sources and private real estate companies. Most of our data were obtained from public data sources, including Statistics Korea, MLIT, the Korea Transport Database, and the Spatial Information Portal. Private real estate companies also publish real estate information online, and we extracted supplementary property information from kbland.kr, land.naver.com, and realty.daum.net websites. The variables collected and assembled for this study are presented in Table 2. The explanatory variables are categorized into five groups, including property characteristics, environmental amenities, local built environment, local demographics, and sales period controls. Descriptive statistics for the listed variables are provided in the Supplementary Information. Variables that were distributed far from a normal distribution were log-transformed. Not all variables in Table 2 were included in the final model because some were excluded in the process of achieving the best model fit.

**Table 2:** Candidate variables.

Category		Variables
Property prices		Actual transaction prices
Property characteristics	Unit-related	Unit size, floor location
	Complex^a^-related	Number of units, Number of apartment buildings, Construction year, Heating type^b^, Parking spaces per unit, Highest floor
Environmental amenities		Street greenness, Road distance to nearest waterfront^c^, Road distance to nearest green space^d^
Local built environment		Road distance to closest subway station, Number of bus stops^e^, Road distance to the central business district (CBD)^f^, Number of top university entrants^g^, Number of high schools^h^
Local demographics		Population, Population density, Adults with higher degrees^i^, Young population ratio^j^, Elderly population ratio^k^, Median age, Sex ratio
Sales period controls		Spring, Fall, Winter

Variables are measured at the administrative level called *Dong*, which is equivalent to a Census District in the US. ^a^A complex consists of several apartment buildings and provides community services such as security and maintenance of shared areas. ^b^Dummy variable: city gas 0 and others 1. ^c^Shortest distance to the nearest river, stream, pond, or seashore by road. ^d^Shortest distance to the nearest park, hill, or mountain by road. ^e^Total number of bus stops within a 400-m radius of the apartment. ^f^Shortest distance to city hall by road. ^g^Number of Seoul National University entrants from high schools within a 5-km radius. ^h^Number of high schools within a 5-km radius. ^i^Number of people with higher degrees / number of people aged 15 years old or over. ^j^Number of people aged less than 15 years old / total population. ^k^Number of people aged 65 years old or over / total population.

We summarize the combination of variables used in our regression analysis and clarify their definitions in Table 3. As noted earlier, considering model fitness, 18 variables are confirmed for a series of hedonic models. The *Variable* column denotes the name of each variable, listed in candidate variables; hence, the *Name* column indicates the name of each variable used in regression analysis. The *Detail* column describes the characteristics of each variable. The structure of the dataset is aligned with existing literature^35,50,57^.

**Table 3:** Definition of explanatory variables.

Variable	Name	Detail
Property characteristics		
Size of the unit	Size	Unit size aggregated in square meters (m^2^)
Floor location	Floor	Apartment’s floor in the building
Number of units	Units	The number of households in an apartment complex
Parking spaces per unit	Parking	The number of parking spaces divided by the number of households
Heating type	Heating	The heating type of each housing: 0 for city gas and 1 for others
Construction year	Year	The construction year of each apartment complex
Environmental amenities		
Road distance to nearest green space	Dist. green	Network distance to the nearest park, hill, or mountain in meters
Road distance to nearest waterfront	Dist. water	Network distance to the nearest river, stream, pond, or seashore in meters
Street greenness	Green index	The degree of street greenness from the pedestrian perspective
Local built environment		
Road distance to closest subway station	Dist. subway	Network distance to the nearest subway station in meters
Number of top university entrants	Top univ.	Number of Seoul National University entrants from high schools within a 5-km radius of apartments
Local demographics		
Sex ratio	Sex ratio	The percentage of the number of males divided by the number of females
Population density	Pop. density	Number of people per square kilometer (km^2^)
Adults with higher degrees	Higher degree	The percentage of the number of people with a higher degree divided by the number of people aged 15 or older
Median age	Medium age	The percentage of the number of people aged 15 to 65 divided by the total population
Seasonality control		
Spring	Spring	Seasonal dummy indicating that a transaction occurred in March, April, or May
Fall	Fall	Seasonal dummy indicating that a transaction occurred in September, October, or November
Winter	Winter	Seasonal dummy indicating that a transaction occurred in December, January, or February

### Analytical procedures

**Figure 3:**
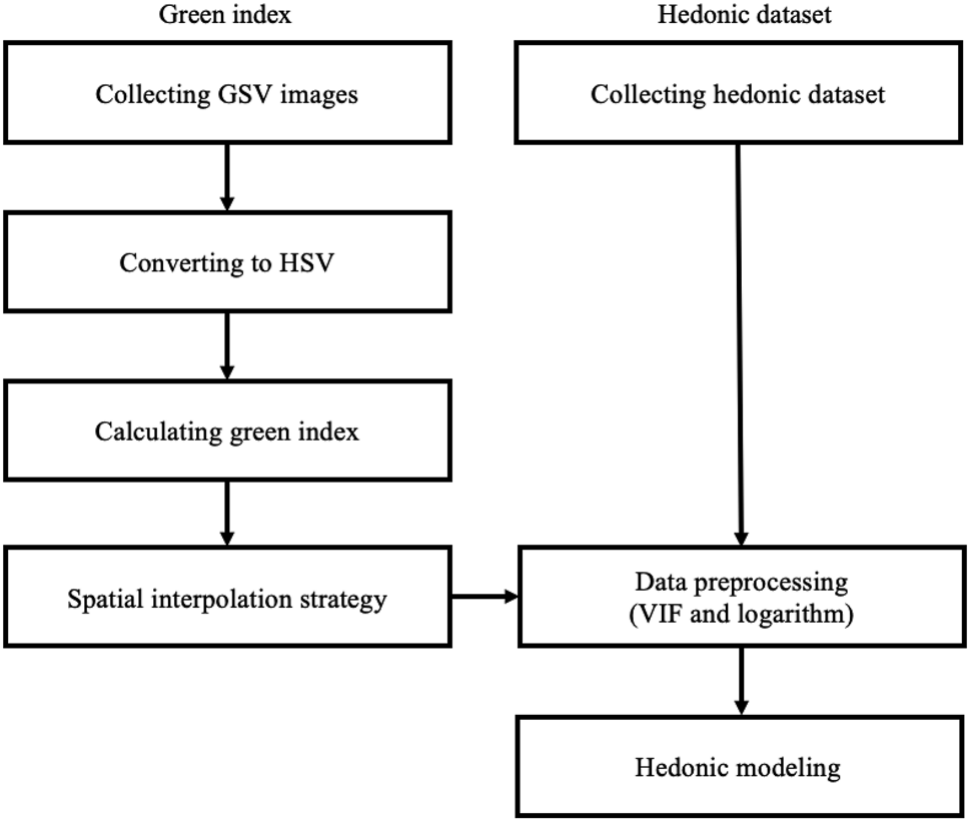
Analysis procedures.

Our analytical procedures are illustrated in Fig. 3. First, we acquire the hedonic data, which includes property characteristics, environmental amenities, local built environment, local demographics, and seasonality controls. Our key variable, green index, is categorized into environmental amenities and obtained referencing GSV images using a pixel-wise approach^16,26^. First, the upper and lower bounds for reflecting natural greenness were employed in this procedure with 306,425 GSV images that fully cover the survey area. Second, we transformed the images to hue, saturation, and value (HSV) color space to calculate the green indices. Third, we employ spatial interpolation, which converts point data into area data, to remedy missing values. Finally, we predict the housing prices using two hedonic models after checking the variance inflation factors (VIF).

### Green index

#### Measuring greenness

**Figure 4:**
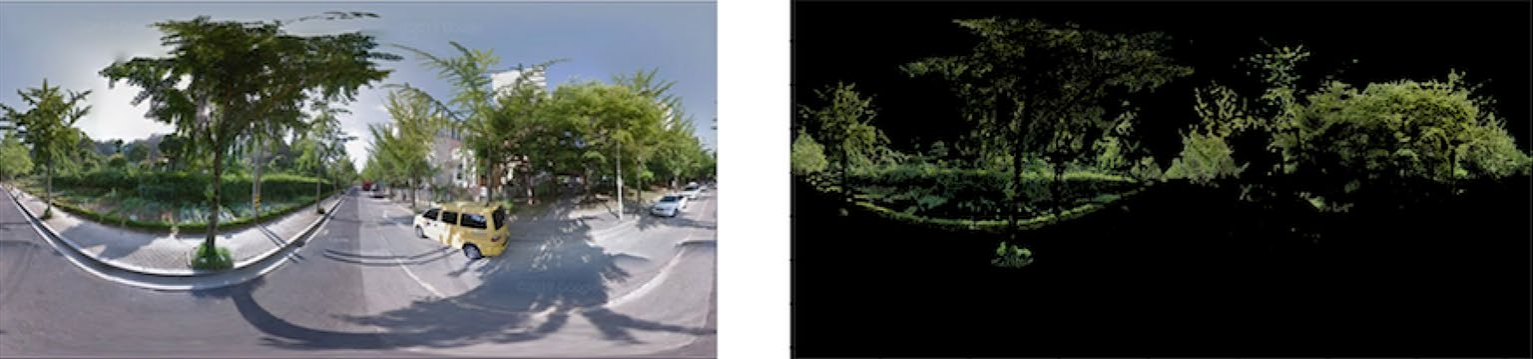
GSV image and its grayscale image (left, original; right, grayscale).

We calculated the extent of greenness for each GSV image employing a four-step process. First, we converted the images from the red, green, and blue color space to HSV color space to make hazy images clear^58,59^. We next defined upper and lower greenness boundaries to precisely capture natural greenery. Considering HSV color scale, the boundary is set by reflecting natural greenness. We then converted each pixel to grayscale. This conversion assigned its own pixel value if the image fell between the boundaries indicating greenness and a value of zero if it did not (Fig. 4). Following this procedure, the green index of each GSV image was calculated as follows:

$$Green \ index={pixel}_{i}/{pixel}_{total}\times 100,$$

where ${pixel}_{i}$ is the number of pixels with a nonzero value and ${pixel}_{total}$ stands for the number of pixels in the image. Therefore, the green index reflects the degree of natural greenness in each image from the pedestrian point of view.

#### Spatial interpolation

Examining the statistical association between street greenery and property prices requires assigning a level of greenness to each housing unit; however, the green index and property prices were recorded as point data. Unlike the area data that are usually available for administrative units, point data rarely match one another, which requires further processing.

**Figure 5:**
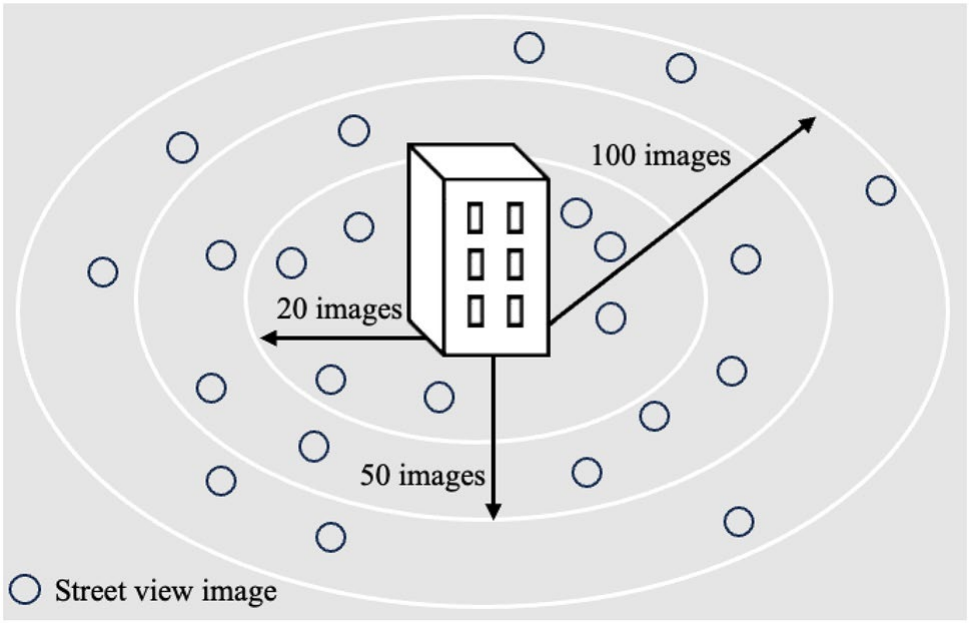
Conceptual illustration of spatial interpolation.

Figure 5 graphically illustrates the detail of our spatial interpolation strategy. Each circle denotes the calculated green index of each GSV image. First, we computed the distances between the target property and all green indices based on housing unit’s latitude and longitude. We then sorted the distances in ascending order and assigned the average value of green indices to individual housing unit by aggregating the nearest images within certain criteria (nearest 20, 50, and 100 images).

Our approach using the spatial interpolation strategy can be a desirable choice for effectively managing point data and/or missing values. Li and Heap^60^ documented that this method is effective for dealing with environmental variables at unsampled sites using data from point observations. Further, its results are free from modifiable areal unit problem, which is often criticized as a source of statistical bias^39,61^. This interpolation strategy is also helpful for enhancing spatial accuracy and has been applied to various disciplines such as identifying potential sites of renewable energy sources^62^ and integrating mobile phone data^63^.

This method covered the target survey area by transforming discrete points into areal information. By using spatial interpolation, our study can explore the association between linear greenness (proximity to green space) and areal greenness (green index). Only latitude and longitude information are required to employ the spatial interpolation strategy. Accordingly, this simple implementation method can be widely applied in various research such as spatial analysis and urban studies.

Street greenery is a local amenity that residents or visitors can enjoy while staying in or passing through an area; therefore, it is more likely that the local amenities include all neighborhood greenery rather than that in the immediate surroundings or the closest images to a property. We plotted image points, assigned each image to calculated green index values, and plotted the individual housing point using the coordinates provided by Google and addresses from the MLIT data (Fig. 2). The average value of green indices in the nearest 20, 50, and 100 images was then assigned to each house [*An alternative method could be to use distance buffers; however, the same number of images was used in this study due to the nonuniform distribution of street images. With distance buffers, some housing units would be assigned no street images, generating missing values. This issue cannot be easily ignored because images in areas close to mountains, hills, and seafronts are particularly rare, indicating that missing values occur nonrandomly. Therefore, we applied the strategy of averaging over specific numbers of images to avoid statistical and practical issues*]. Average coverage referencing the number of images is approximately an 80-, 110-, and 150-m radius from each dwelling, respectively. This coverage crudely accords with buffer sizes previously used to investigate the impact of green space^37^ and in the community conception proposed by Donaldson^64^.

### Estimating housing prices

Hedonic modeling has been widely used to evaluate property prices and has its roots in consumer theory^65^ and Rosen’s hedonic price model^66^, which asserted that housing differs from other consumer goods and that real estate properties should be treated as goods sold as a package of inherent attributes^66,67^. Numerous studies have used hedonic modeling to statistically examine the relationship between property prices and various properties and local characteristics^67–70^. We specify a standard hedonic model in log-linear form as follows:

1 $${{\text{ln}}\,p}_{i}=\alpha +{\sum\limits }_{j=1}^{J}{\beta }_{j}{x}_{ij}+{\sum\limits }_{k=1}^{K}{\theta }_{k}{Q}_{ik}+{e}_{i},$$

where ${p}_{i}$ is the per-square-meter price of each house $i$; $J$ is the number of explanatory variables, such as housing characteristics, local amenities, and local demographic characteristics (${x}_{ij}$); $K(=3)$ is the number of seasonal dummies (${Q}_{ik}$). ${\beta }_{j}$ and ${\theta }_{k}$ are regression coefficients estimated using the ordinary least squares method; and ${e}_{i}$ is the residual.

In addition to housing characteristics, the prices of nearby housing could influence the price of a housing unit^25^. Accordingly, the spatial lag hedonic model was employed to account for spatial dependence^71^, incorporating a spatial lag term ($Wy$) into the hedonic price model as follows:

2 $$p=\rho Wp+{\rm X}\beta +\varepsilon ,$$

where $p$ is the $n\times 1$ vector of each housing price on a logarithmic scale; $\rho$ is the spatial lag parameter, minimizing the root mean square error in the range $(-1, 1)$; $W$ is the spatial weights matrix whose dimension is $n\times n$ in a row-standardized form; ${\rm X}$ is the $n\times k$ matrix of hedonic variables; and $\beta$ is the $k\times 1$ vector of regression coefficients. Finally, $\varepsilon$ is the $n\times 1$ vector of residuals, which are assumed to be homoscedastic, independent across observations, and normally distributed.

The spatial weight matrix is defined as follows:

$${W}_{rs}=\left\{\begin{array}{cc}1/{d}_{rs}& \mathrm{ for} \ {d}_{rs}<D\\ 0 & {\text{otherwise}}\end{array}\right. ,$$

where $r$ and $s$ represent the location of each house with its longitude and latitude; ${d}_{rs}$ is the distance between two properties $r$ and $s$ based on their latitudes and longitudes; and $D$ is the distance band defined by 1. Finally, Eq. (2) can be revised as follows^35^:

3 $$\left(I-\rho W\right)y={\rm X}\beta +\varepsilon .$$

Because the dependent variable $y$ in Eq. (2) would be jointly explained, the spatial weights matrix $W$ could be considered as endogenous which might lead to biased estimation^35,72^. To remedy this, we further conducted our regression analysis based on Eq. (3).

## Results and discussion

### Analysis results

We summarize the coefficients of our hedonic models in Tables 4 and 5. We conducted our analyses using three different numbers of GSV images for spatial interpolation (i.e. 20, 50, and 100) and we only report the results of using 50 images per observation; the other two aggregation strategies still produced fairly good and consistent results. Furthermore, VIF was calculated for various combinations of explanatory variables prior to regression analysis to assess potential multicollinearity. VIF ranged between 1.06 and 1.80 for the reported models. Our models in Tables 4 and 5 have similar predictive power, accounting well for the variance in housing prices, with significant *F*-statistics and all regression coefficients in the final models are significant at the 1% level.

**Table 4:** Results from ordinary least squares models.

Obs. = $\mathrm{52,644}$	(1)	(2)	(3)
Property characteristics			
Size	$0.012$^‡^	$0.012$^‡^	$0.012$^‡^
Floor	$0.005$^‡^	$0.005$^‡^	$0.005$^‡^
Units	$8.4\times {10}^{-5}$^‡^	$8.6\times {10}^{-5}$^‡^	$8.5\times {10}^{-5}$^‡^
Parking	$0.103$^‡^	$0.103$^‡^	$0.102$^‡^
Heating	$0.160$^‡^	$0.158$^‡^	$0.158$^‡^
Year	$0.013$‡	$0.013$^‡^	$0.013$^‡^
Environmental amenities			
Dist. green	$-0.008$^‡^	–	$-0.008$^‡^
Dist. water	$-0.012$^‡^	$-0.011$^‡^	$-0.012$^‡^
Green index	*–*	$-0.007$^‡^	$-0.007$^‡^
Local built environment			
Dist. subway	$-0.072$^‡^	$-0.072$^‡^	$-0.072$^‡^
Top univ.	$0.005$^‡^	$0.005$^‡^	$0.005$^‡^
Local demographics			
Sex ratio	$-0.006$^‡^	$-0.007$^‡^	$-0.007$^‡^
Pop. density	$2.7\times {10}^{-6}$^‡^	$2.6\times {10}^{-6}$^‡^	$2.7\times {10}^{-6}$^‡^
Higher degree	$0.006$^‡^	$0.006$^‡^	$0.006$^‡^
Medium age	$0.003$^‡^	$0.003$^‡^	$0.003$^‡^
Seasonality control			
Spring	$0.016$^‡^	$0.013$^‡^	$0.016$^‡^
Fall	$0.073$^‡^	$0.078$^‡^	$0.073$^‡^
Winter	$0.051$^‡^	$0.050$‡	$0.050$‡
Constant	$-16.393$^‡^	$-15.974$^‡^	$-15.989$^‡^
* F*-statistics	$\mathrm{6,960}$^‡^	$\mathrm{6,950}$^‡^	$\mathrm{6,590}$^‡^
RMSE	$0.315$	$0.315$	$0.315$
Adjusted *R*^*2*^	$0.692$	$0.692$	$0.693$

^‡^Indicates significance at the 1% level.

**Table 5:** Results from spatial lag models.

Obs. = $\mathrm{52,644}$	(1)	(2)	(3)
Property characteristics			
Size	$0.012$^‡^	$0.012$^‡^	$0.012$^‡^
Floor	$0.004$^‡^	$0.004$^‡^	$0.004$^‡^
Units	$7.8\times {10}^{-5}$^‡^	$7.8\times {10}^{-5}$^‡^	$7.8\times {10}^{-5}$^‡^
Parking	$0.086$^‡^	$0.086$^‡^	$0.085$^‡^
Heating	$0.098$^‡^	$0.097$^‡^	$0.097$^‡^
Year	$0.013$^‡^	$0.013$^‡^	$0.013$^‡^
Environmental amenities			
Dist. green	$-0.005$^‡^	–	$-0.005$^‡^
Dist. water	$-0.010$^‡^	$-0.010$^‡^	$-0.010$^‡^
Green index	–	$-0.002$^‡^	$-0.003$^‡^
Local built environment			
Dist. subway	$-0.051$^‡^	$-0.051$^‡^	$-0.051$^‡^
Top univ.	$0.003$^‡^	$0.003$^‡^	$0.003$^‡^
Local demographics			
Sex ratio	$-0.003$^‡^	$-0.003$^‡^	$-0.003$^‡^
Pop. density	$1.9\times {10}^{-6}$^‡^	$1.9\times {10}^{-6}$‡	$1.9\times {10}^{-6}$^‡^
Higher degree	$0.005$^‡^	$0.005$^‡^	$0.005$^‡^
Medium age	$0.005$^‡^	$0.005$^‡^	$0.005$^‡^
Seasonality control			
Spring	$0.015$^‡^	$0.013$^‡^	$0.015$^‡^
Fall	$0.063$‡	$0.066$^‡^	$0.063$^‡^
Winter	$0.047$^‡^	$0.046$^‡^	$0.047$^‡^
Constant	$-19.697$^‡^	$-19.541$^‡^	$-19.550$^‡^
* F*-statistics	$\mathrm{6,920}$^‡^	$\mathrm{6,900}$^‡^	$6,540$^‡^
RMSE	$0.292$	$0.292$	$0.292$
Adjusted *R*^*2*^	$0.691$	$0.690$	$0.691$

^‡^Indicates significance at the 1% level.

### Street greenery and housing price

**Figure 6:**
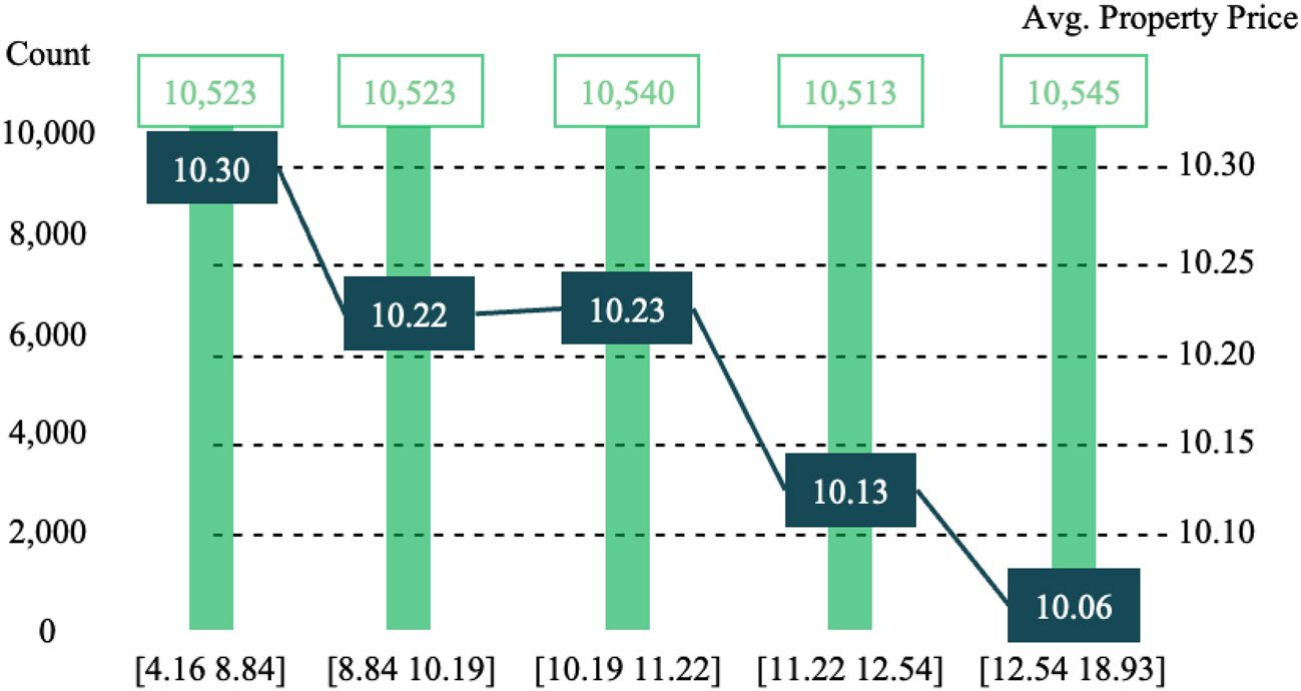
Housing prices and green index. Dark boxes indicate the average housing prices within the range of the green index band. The top boxes present the number of observations within the range.

Our results indicate that the green index is significantly negatively associated with housing prices. The quality of greenery could be the reason for the negative signs of street greenery. The images alone do not provide a quality measure but higher exposure to scenic views could contribute to the quality of environmental amenities^10^; thus, we grouped the level of greenness to examine the association between greenness intervals and housing prices. Figure 6 presents the average value of log-transformed housing prices per square meter by street greenery levels. We divided the green index into five bands, assigning similar numbers of observations into each band [*More or a smaller number of bands and different grouping strategies were also adopted but we show only the five band results with similar numbers of observations because these presented consistent results*]. The findings reveal that housing prices tend to have an inverse relationship with street greenery levels and no preferred greenery levels are evident in terms of financial premium, which supports the robustness of our regression results (Tables 4 and 5).

Figure 6 clearly reveals that greener streets do not command financial premiums for housing prices. The sign of the association between housing price and street greenery in this case study site requires careful interpretation. Street’s green amenities generally form parts of the street landscaping in urban areas like Busan. Therefore, more trees and shrubs indirectly indicate more streets, which could be considered a dis-amenity to housing assets. In Panduro and Veie’s^27^ classification of green space, the street greenery in the images is a green buffer, which explains the different association observed from another study that used the same data source in New York^16^. Furthermore, this study examined residential units, whereas Yang et al.^16^ used commercial properties. For commercial properties, pedestrian and traffic flow would be value-adding, while those living in residential units may not appreciate a dense road and path network in neighborhoods.

Adding more complexity, longstanding and strongly rooted trees tend to have more leaves, translating to more green pixels in GSV images. In Busan, the correlation between construction year and green index is significantly negative (Table 6), implying that newer apartments tend to have less surrounding street greenness. When the construction of an apartment complex is planned, roads and paths adjacent to the construction site are modified to accommodate an expected increase in auto and pedestrian traffic and changed land use. The shape and width of existing roads and paths are amended, and street landscaping is renewed to reflect those changes. Therefore, recently constructed apartments are more likely to be surrounded by young and newly planted trees and shrubs. As such, we can infer that the negative association between street greenery and housing prices does not necessarily suggest that people do not appreciate green streets but that structural issues are involved. Therefore, we conclude that street-level greenness is indeed associated with housing prices, but the sign of this association reflects the local context of the study area.

**Table 6:** Pairwise correlation between construction year and green index.

	Green index (20)	Green index (50)	Green index (100)
Correlation coefficient	$-0.073$^‡^	$-0.116$^‡^	$-0.126$^‡^

^‡^Indicates a significance at the 1% level.

### Other amenities, characteristics, and housing price

Two green amenity variables, distance to green space (*Dist. green* in Tables 4 and 5) and the green index, were separately and jointly introduced into the models, revealing that the model containing *Dist. green* and the *Green index* predicted housing prices better than or similar to the models including only one greenery variable. Furthermore, our two green amenity variables had different associations with housing prices. Since the former variable has a negative marginal contribution to housing price, we can infer that residents prefer green space that is near their homes. The latter variable also has a negative marginal effect, implying that street greenness has the opposite association with housing prices compared with green space. This result indicates the different functions of the two green amenities. As previously noted, street greenery cannot be considered as a purely green amenity in Busan’s local contexts. It could be a sign of an old neighborhood or a buffer connected to busy roads, while green space indicates spaces where urbanites can relax, have social interactions in comfort, and enjoy a cleaner environment^18,73,74^. Therefore, the two greenery variables present a complementary relationship rather than a substitute in both statistical and contextual terms.

Two environmental amenity variables other than the green index exhibited significant negative associations with housing prices, implying that urbanites are willing to pay higher prices to live closer to the waterfront and/or green spaces. Research has demonstrated that urban green space has a strong positive association with housing prices because of its aesthetic value and provision of a clean environment^7,17,73^. Benson et al.^10,19^ showed that a scenic view is a significant factor in predicting housing prices. The authors categorized ocean views by the level of obstruction, finding that better views added a higher premium to housing prices. In this context, distance to the waterfront could be an adequate proxy in our models. Other studies have also noted that residential and commercial properties have a significantly positive relationship with the surrounding landscape and facilities such as rivers, lakes, and other water bodies in tourist cities^75,76^. As such, our empirical findings corroborate the results in existing literature.

Other notable variables are evident in the results tables. Housing characteristics have positive effects on housing prices, and larger units on higher floors in residential properties with plenty of parking spaces tend to command higher prices, aligning with previous case study results^35^. Moreover, construction year is also significantly associated with property prices, implying that newly built apartments are more attractive^77,78^. Property size indicates a significantly positive coefficient, implying that residents also prefer spacious dwellings over small living environments^79^. The negative association with distance to the closest subway station and the positive association with top university entrants indicate the benefits of transportation access and educational environment for willingness to pay higher housing prices.

### Managerial and practical policy implications

#### Greenness in urban areas

Urban greenness is emphasized by United Nations Development Program in Sustainable Development Goal 11: Sustainable cities and communities, particularly in relation to universal access to green and public spaces^80^. With recognition of such importance, there have been an effort to build valuation toolkits of urban green infrastructure, but not many of them comprehensively assess urban green infrastructure yet^81^. We adopted proximity to green space and the visual quality of street-level greenness as key components of greenness in urban areas; both are important components for building sustainable communities. When policymakers only focus on single indicator to assess urban greening, it may lead to biased decisions resulting in unbalanced development of the ecological environment.

Accordingly, the complementary relationship between our two greenness indicators provides valuable insights for policymakers and urban planners to improve street-level greenness in addition to green accessibility. We argue that the negative association between the street-level greenness and housing prices in Busan, South Korea should not be misunderstood as street greenness negatively impacting housing prices. Rather, our findings imply that policymakers should encourage the rapid recovery work of street-level greenness around newly built structures to facilitate resilient green surroundings.

#### Ecological transition

Since the construction of ecological civilization is crucial for promoting sustainable development^82^, residents’ perception of ecological transition must be improved. As a vital means for advancing sustainable communities, ecological transition to achieve change complied with the criteria for environmental sustainability is worthy of noting^83,84^. Previous studies have noted the importance of urban greenness in increasing environmental awareness and well-being^85–87^ In addition, ecological transition is closely associated with urban greenness and must include citizens’ participations^88^.

In this context, urban designers and related policymakers must broaden political views to consider additional dynamics related to civil society, social movements, and consumer preferences. To support these public professionals, our work demonstrates that street-level green visibility and proximity to open green space represent complementary environmental amenities that are closely related to housing prices. This empirical evidence can be regarded as presenting an additional criterion for ecological transition that can inform policymaking and urban design and promote the active involvement of residential communities.

#### Sustainable education

According to Geels^89^, private actors have no immediate incentive to address sustainability; therefore, public authorities and civil communities are the primary drivers of sustainability transitions. Conversely, in the 2020 McKinsey US consumer sentiment survey^90^, when consumers were asked if they cared about environmentally sustainable products, they overwhelmingly agreed. This indicates that sustainable education has improved consumers’ perceptions regarding sustainable communities.

We also found that residents in our case study site would be willing to pay a premium for housing that is near natural environmental amenities, whereas green visibility was negatively associated with residents’ preference due to the unique context of residential area development in Busan. Such construction may diminish the role of sustainable education as a significant factor for sustainable cities and development. Since green space positively influences the academic environment (i.e. the sense of belonging and academic performance)^91,92^, the significance of the visual quality of urban greenness is further emphasized. Accordingly, to encourage sustainable education, the visual quality of street-level greenness must be improved around schools to produce sustainable education environments, which aligns with Sustainable Development Goal 4: Quality Education by United Nations Development Programme^93^.

## Conclusion

It is well known that environmental amenities are valued in the housing market. This study demonstrated how GSV images can be used to reveal the role of such amenities in predicting housing prices within a metropolitan area. Although these images do not have a long history of usage in this field of study, we showed that street greenness has a complementary relationship with other environmental amenity variables in the case study site. Specifically, visual street greenness possibly had a negative association with housing prices due to the contemporary strategy of constructing residential complexes. This indicates that policy makers and practitioners should consider various aspects of natural environments when designing and implementing development and/or preservation plans. Further, our findings emphasize the importance of local context when determining how environmental amenities are recognized and reflected in local housing markets. Without thorough understanding of local context, analysis results do not reveal actual meanings and implications by themselves leading to failure of timely and proper policy development.

Methodologically, this study contributes to the real estate and urban planning literature by developing a three-step approach to quantify street greenness near properties with novel and useful evidence. GSV images do not cover all of the streets in certain areas. Our interpolation strategy is useful for such cases. In particular, only location information such as latitude and longitude was required for this method; thus, our approach expands the practical potential of images taken at various points. Although spatial interpolation is commonly used in the Geographic Information System field, it has not yet been well used in other fields of study. Moreover, our method provides a way to overcome scale problems so it can work effectively on the studies where mixed scale data or point data should be in use. As various types of big data become more available, our method could be one of useful approaches to handle the mismatch of data scales.

GSV images can be a useful source for objectively measuring visibility from the pedestrian perspective; however, this approach has inherent limitations because images are commonly collected from vehicles, resulting in some irrelevant images and/or uneven distribution of images. In addition, GSV images are only captured at specific time points; hence, recording data (e.g., videos that include continuous scenery and landscapes) could be more informative. Accordingly, future studies can consider various data sources to investigate the impact of environmental amenities on residential communities. Wellbeing and happiness could be a good example as not much of urban greenness studies rigorously worked on the association between urban trees and wellbeing^87^. By doing so, future research can capture the diversity of environmental amenities more accurately to contribute to urban planning and policymaking.

### Supplementary Information

Supplementary Table S1.

## Data Availability

The datasets used and/or analyzed during the current study are available from the corresponding author on reasonable request.
