# Supplementary material for: Assessment of street-level greenness and its association with housing prices in a metropolitan area
Source: Sci Rep. 2023 Dec 19;13:22577. doi: 10.1038/s41598-023-49845-0 (PMC10730891; doi:10.1038/s41598-023-49845-0)
Supplement: Supplementary file 1 — Supplementary Table S1. [file 41598_2023_49845_MOESM1_ESM.docx]

|  | Mean | Std. | Min. | Max. |
| --- | --- | --- | --- | --- |
| Housing price^a^ | $10.187$ | $0.568$ | $6.908$ | $12.934$ |
| **Property Characteristics: Unit-related** | | |  |  |
| Size | $77.820$ | $28.916$ | $12.488$ | $269.680$ |
| Floor | $11.819$ | $8.201$ | $-1.000$ | $77.000$ |
| **Property Characteristics: Complex-related** | |  |  |  |
| Units | $937.087$ | $885.506$ | $4.000$ | $5,239.000$ |
| Buildings | 9.227 | 8.910 | 1.000 | 77.000 |
| Year | $2003.496$ | $10.153$ | $1969$ | $2019$ |
| Heating | $0.093$ | $0.291$ | $0.000$ | $1.000$ |
| Parking | $1.101$ | $0.619$ | $0.000$ | $77.000$ |
| Highest floor | 23.249 | 10.347 | 2.000 | 84.000 |
| **Environmental Amenities** | | |  |  |
| Dist. Green^a^ | $7.277$ | $2.222$ | $0.808$ | $10.714$ |
| Dist. Water^a^ | $6.268$ | $1.181$ | $-0.170$ | $8.601$ |
| Green Index | $10.733$ | $2.098$ | $4.163$ | $18.927$ |
| **Local Built Environment** |  |  |  |  |
| Dist. Subway^a^ | $6.892$ | $1.016$ | $3.366$ | $9.978$ |
| Bus stop | 18.105 | 10.840 | 0.000 | 63.000 |
| Dist. CBD | 237,377.312 | 192,493.328 | 243.860 | 398,856.132 |
| Top Univ. | $11.179$ | $6.442$ | $0.000$ | $27.000$ |
| High school | 14.274 | 7.295 | 0.000 | 30.000 |
| **Local Demographics** | | |  |  |
| Population | 25,888.373 | 14,125.007 | 1,208.000 | 83,116.000 |
| Pop. Density | $13,220.993$ | $10,687.626$ | $1.003$ | $118,181.818$ |
| Higher Degree | $30.303$ | $9.847$ | $10.356$ | $61.289$ |
| Young population | 12.120 | 4.121 | 3.151 | 26.285 |
| Elderly population | 16.325 | 4.455 | 5.712 | 33.292 |
| Medium Age | $42.603$ | $3.637$ | $32.700$ | $55.4000$ |
| Sex Ratio | $95.750$ | $4.656$ | $81.024$ | $124.508$ |
| **Sales Period Control** | | |  |  |
| Spring | $0.215$ | $0.411$ | $0.000$ | $1.000$ |
| Fall | $0.343$ | $0.475$ | $0.000$ | $1.000$ |
| Winter | $0.243$ | $0.429$ | $0.000$ | $1.000$ |

**Table S1.** Descriptive statistics of the variables. ^a^ This variable is log-transformed.
